# Atlantification advances into the Amerasian Basin of the Arctic Ocean

**DOI:** 10.1126/sciadv.adq7580

**Published:** 2025-02-21

**Authors:** Igor V. Polyakov, Andrey V. Pnyushkov, Matthew Charette, Kyoung-Ho Cho, Jinyoung Jung, Lauren Kipp, Morven Muilwijk, Laura Whitmore, Eun Jin Yang, Jaeill Yoo

**Affiliations:** ^1^ International Arctic Research Center and College of Natural Science and Mathematics, University of Alaska Fairbanks, Fairbanks, AK 99775, USA.; ^2^ International Arctic Research Center, University of Alaska Fairbanks, Fairbanks, AK 99775, USA.; ^3^ Woods Hole Oceanographic Institution, 266 Woods Hole Road, Woods Hole, MA 02543, USA.; ^4^ Korea Polar Research Institute, Incheon, Korea.; ^5^ Rowan University, 201 Mullica Hill Road, Glassboro, NJ 08028, USA.; ^6^ Norwegian Polar Institute, Fram Centre, Tromsø, Norway.

## Abstract

Atlantification—the northward inflow of anomalous waters and biota from the Atlantic into the polar basins—has wide-ranging climatological ramifications. We present previously unknown observational evidence that the atlantification processes are strengthening in the eastern Eurasian Basin. The primary example is the diminishing sea ice, which is related to a powerful ocean-heat/ice-albedo feedback, which accelerates sea-ice losses. Furthermore, we observe that atlantification is extending far beyond the Lomonosov Ridge into the Makarov Basin of the Arctic Ocean where upper ocean ventilation creates a new and unique ecological environment. The eastern part of the Siberian Arctic Ocean is still strongly stratified, but the atlantification-driven shoaling of warm, salty, and nutrient-rich intermediate waters already has important ecological consequences there. Disentangling the role of atlantification in multiple and complex high-latitude changes should be a priority in future modeling and observational efforts.

## Teaser

Contrary to the global climate model projections, atlantification has already spread into the Amerasian Basin of the Arctic Ocean.

## INTRODUCTION

The primary indicators of global climate change over the past few decades have been the significant decreases in sea ice and the warming of the Arctic’s atmosphere and ocean [e.g., (*1*)]. An important element of this high-latitude climate change has been the northward advection of anomalous subarctic Atlantic- and Pacific-origin waters and biota into the polar basins, known as atlantification and pacification, respectively (*2*).

**Fig. 1.**
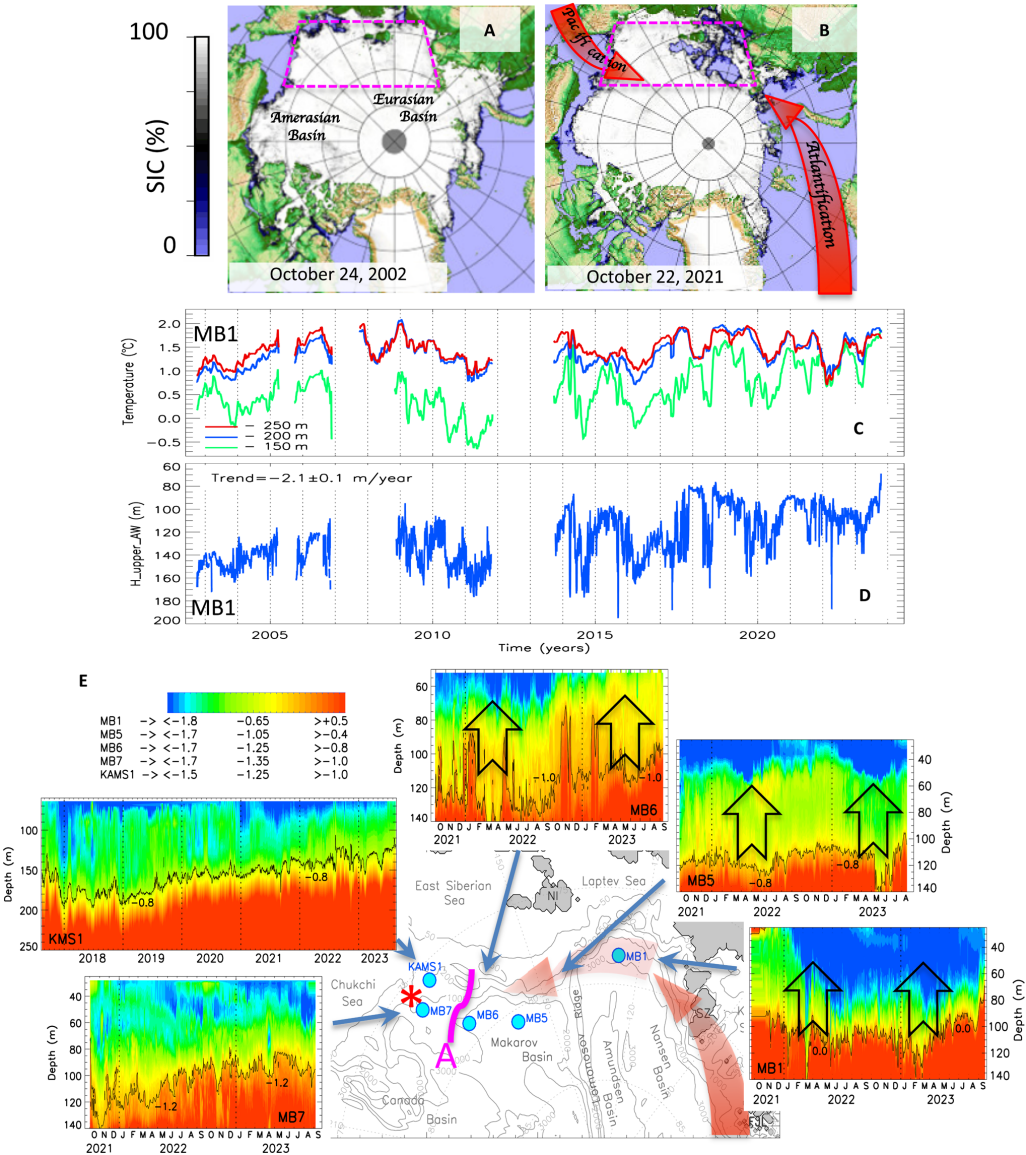
Advancement of atlantification into the Arctic Ocean interior. (**A** and **B**) Examples of Arctic sea-ice concentration (SIC, %) during the start of the freezing season from the early 2000s (A) and late 2010s (B). The absence of sea ice in the Siberian Arctic Ocean indicates that atlantification and pacification are swiftly moving into the Arctic Ocean’s interior along the edge of the Siberian shelf. Siberian Arctic Ocean is indicated by magenta boxes. (**C** and **D**) Composite 2002–2023 monthly mean time series of (C) water temperature and (D) the upper AW boundary (defined by the 0°C isotherm) from the mooring site MB1, eastern Eurasian Basin of the Arctic Ocean. White segments indicate missing data. (**E**) Mooring temperature records showing different stages of development of atlantification along the Siberian slope. Western moorings MB1, MB5, and MB6 show deep (>100 m) ventilation in winter season (schematically indicated by arrows), whereas eastern moorings MB7 and KAMS1 document shoaling of the AW but no deep winter ventilation (each panel’s isotherms, displayed as black lines, make these patterns easier to see). The boundary between these regions is marked by a magenta curve and A. The position of station P used in Fig. 4 is indicated by the red sign “*.”

The warming of salty [salinity just under 35 psu (practical salinity unit)] and warm (temperature, >0°C) Atlantic water (AW), historically located at 150- to 800-m depth, began in the 1970s (*2*). However, the first evidence of strong atlantification was found in the 1990s when a warm (anomalies up to 1°C) pulse of the AW entered the Nansen Basin (*3*) (see geographical notations in Fig. 1). Following the major deep basin margins in a counterclockwise direction, it began its topography-controlled journey into the ocean interior, arriving in the Makarov Basin by 1993 and the Canada Basin by 2000 (*4*, *5*). A second (warmer by ~0.2°C) AW pulse entered Fram Strait in 1999 (*6*) and advanced into the eastern Eurasian Basin by 2004 (*7*). Data-constrained regional coupled sea ice-ocean model experiments suggested that part of this pulse may cross the Lomonosov Ridge since AW property changes are observed to spread into the Makarov Basin and then into the Canada Basin (*8*). Observations from the 1990s and 2000s showed a shoaling of the AW upper boundary by about 40 to 75 m relative to climatology (*9*–*11*). Despite these major changes in the AW layer, they had little effect on the upper ocean and sea ice because of the strong halocline overlaying the AW, which functioned as a barrier for AW heat flux because of its strong stratification.

In the 1970s, the Eurasian Basin halocline began to weaken (*9*, *11*) and, over three decades, nearly 30% of the water column stability was lost (*11*). By the mid-2010s, this weakening combined with the AW shoaling prompted a transition to a new ocean state: one without the presence of a strong halocline layer where heat can be transferred vertically to the sea ice (*12*). This has substantially reduced the regional rates of winter sea-ice formation and contributed to the loss of sea-ice cover along the AW pathway in the eastern Eurasian Basin (*12*). Furthermore, oceanographic surveys from 2001 to 2020 demonstrated that the sea-ice thickness anomalies caused by atlantification persist across the Arctic region and are prevalent along the entirety of the Transpolar Drift (*13*).

These changes in the eastern Eurasian Basin should result in a powerful positive ocean-heat/ice-albedo feedback mechanism, as hypothesized in (*14*). In this feedback, increased winter ventilation of the ocean interior enhances the release of AW heat to the sea surface, contributing to sea-ice loss. Thinner sea ice melts faster the following summer, triggering the ice-albedo feedback, decreasing sea ice even more, and delaying sea-ice formation next fall. The reduced sea-ice extent during the subsequent freezing season promotes faster sea-ice production, more vigorous inertial oscillations, and related upper-ocean shear, all of which increase the ventilation of the AW (*15*).

After 2007, atlantification was increased by the decadal mode of variability brought on by the cyclonic decadal-scale atmospheric regime associated with weakened anticyclonic winds over North America and enhanced cyclonic winds over Eurasia (*16*). These physical changes altered nutrient fluxes and high-latitude primary production over recent years (*17*, *18*) and have led to shifts in species composition and food web structure (*19*). Through a combination of weakened nutrient, temperature, and light limitation, it is thought that up to 26% of the productivity increases in the Barents Sea could be due to climate-driven atlantification of the Arctic Ocean (*20*).

Strong topographic control over the AW circulation suggests that strong atlantification will remain in the Eurasian Basin. For instance, CMIP-6 model projections indicate that it is unlikely to extend far into the Amerasian Basin (*21*). Moreover, freshening of the upper Canada Basin in recent decades resulted in a regional strengthening of the stratification [e.g., (*22*–*25*)], which mitigates the effects of atlantification. Here, we show results that directly contradict this hypothesis. We present previously unknown observational evidence that the atlantification processes are strengthening in the eastern Eurasian Basin and are extending far beyond the Lomonosov Ridge into the Makarov Basin of the Arctic Ocean (see the map in Fig. 1E with geographical notations). In addition, we argue that mixing of halocline waters creates a unique ecological environment in the eastern Makarov Basin.

## RESULTS

### Atlantification of the Eurasian Basin

More than two decades of mooring data from the eastern Eurasian Basin reveal that the AW core (identified by the maximum temperature at 200 to 250 m; Fig. 1C) is not warmer in recent years than it was in 2007–2008. However, the temperature at 150 m—the level traditionally considered the upper boundary of AW—approached that of the AW core (compare water temperature difference at 250 and 150 m in 2003 to 2007 of 0.77 ± 0.01°C and in 2020 to 2023 of 0.40 ± 0.01°C; Fig. 1C). There is evidence of substantial shoaling of the AW upper boundary from more than 160 m in 2002 to (monthly mean) 65 m in 2023 (Fig. 1D). This new upper limit is well within the depth of regional winter ventilation caused by cooling and salt rejection in the upper ocean during sea-ice formation (*26*). These changes signal a further strengthening of the tendency toward a fundamentally new Eurasian Arctic Ocean, one with no permanent halocline layer and enhanced winter entrainment of AW heat toward the sea ice, first documented in (*12*).

**Fig. 2.**
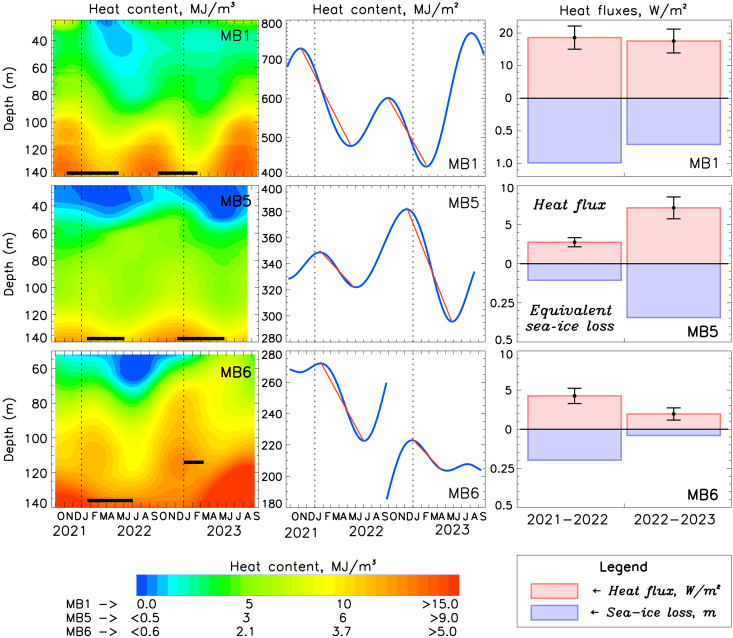
Sea-ice losses due to oceanic heat along the Siberian slope in 2021 to 2023. (**Left**) Depth versus time sections of the annual component of heat content *Q*. Annual components are obtained via band-pass filtering using wavelet transforms. Horizontal black segments identify the depth of seasonal ventilation (used to calculate vertically integrated *Q*); start and end dates of the ventilation seasons are shown by the ends of these segments. (**Middle**) Time series of vertically integrated heat content (blue) and trends of seasonal upper ocean-heat content change (red). The boundaries of the winter ventilation seasons were defined using the maxima and minima of the vertically integrated *Q*. The MB6 record’s time series discontinuity is attributed to the varying depths of integration used for the 2021–2022 and 2022–2023 periods. (**Right**) The ventilation seasons’ trends in *Q* define the rate of change of *Q* over time, which is equivalent to the divergent heat flux δ*F*_h_ (red bars; errors are shown by black segments). Equivalent sea-ice thickness losses are shown as blue bars.

This recent deep winter ventilation down to 140 m is particularly evident in the 2-year (2021 to 2023) record of water temperature measured from the MB1 mooring (Fig. 1E), but it is particularly well illustrated by the annual component of heat content $Q=\rho_{w}c_{P}\left(\theta -\theta_{\text{fr}}\right)$, where θ is the potential temperature, θ_fr_ is the freezing temperature, ρ_w_ is the water density, and *c*_P_ is the specific heat of seawater (Fig. 2 and fig. S1; for details of *Q* definition, see the Supplementary Materials). The time difference of vertically integrated *Q* offers a measure of divergent heat flux δ*F*_h_ during the winter ventilation season [e.g., (*12*)]. For winters of 2021–2022 and 2022–2023, δ*F*_h_ = 18.6 ± 3.5 and 17.6 ± 3.7 W/m^2^ (positive δ*F*_h_ indicates an upward heat flux), respectively. These values greatly exceed the 8.0 W/m^2^ derived by averaging estimates of δ*F*_h_ from all moorings in the eastern Eurasian Basin in 2013 to 2018 and are statistically indistinguishable from the highest value of δ*F*_h_ = 20.6 ± 6.8 W/m^2^ inferred from the 2017–2018 record (*14*). The equivalent losses of sea-ice thickness resulting from a reduced rate of winter ice formation are of the order of 0.7 to 1.0 m, a sea-ice decrease that greatly exceeds estimates of up to 0.4 m from the early 2010s (*12*).

**Fig. 3.**
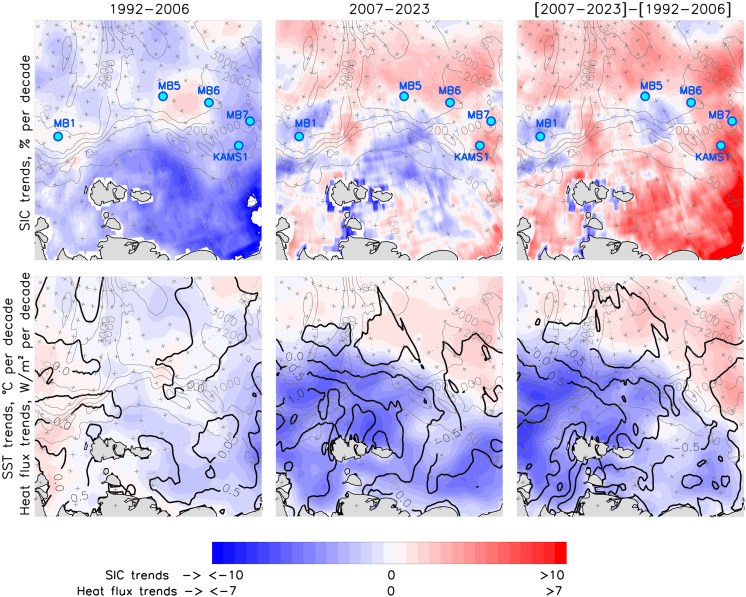
Changes in sea-ice concentration and air-sea interactions caused by the ocean-heat/ice-albedo feedback mechanism. (**Top row**) Satellite sea ice concentration trends for 15 October to 15 November. Mooring locations are indicated by blue dots. (**Bottom row**) ERA5 reanalysis net air-sea heat flux trends (blue color indicates increasing rates of heat release from the ocean) for the same season (color) and sea surface temperature (SST) trends for 15 September to 15 October (thick black lines).

The changes in the eastern Eurasian Basin after 2007 provide clear indications of the ocean-heat/ice-albedo feedback’s fingerprint along the continental slope of the eastern Eurasian Basin, which is the primary conduit of atlantification. In winter, this feedback is evident by increased oceanic heat fluxes and equivalent sea-ice losses (as discussed in the previous paragraph). In summer, it is linked to heat accumulation as shown by elevated regional sea surface temperature [a good proxy for upper ocean-heat content; (*2*)], which results in increased heat fluxes from the ocean further reducing sea ice during the fall freezing season (Figs. 1, A and B, and 3 and figs. S2 and S3). It is established that in the Barents Sea, diminished sea ice during one winter season reduces the likelihood of sea-ice formation the following year (*27*–*30*). The continued regional sea-ice losses along the Siberian slope despite the hiatus of sea-ice loss in the Amerasian Basin after 2007 (Fig. 3) (*16*) provide evidence of the high efficacy of this feedback mechanism, further confirming enhanced coupling between atmosphere, sea ice, and ocean in the Siberian Arctic (Fig. 3 and figs. S2 and S3) (*15*, *16*).

### Advancement of atlantification into the Amerasian Basin

Mooring records (MB5 and MB6) from 2021 to 2023 in the Makarov Basin, located on the Amerasian side of the Lomonosov Ridge, show a notably similar pattern of winter deep (114 to 140 m) ventilation (associated with cooler temperatures progressing from the surface into the interior) compared with the eastern Eurasian Basin (Figs. 1 and 2). For the winters of 2021–2022 and 2022–2023, for example, the inferred estimates of divergent heat flux δ*F*_h_ are 2.7 ± 0.6 and 7.1 ± 1.4 W/m^2^ for the MB5 mooring location and 4.2 ± 1.0 and 1.0 ± 0.3 W/m^2^ for the MB6 mooring location, respectively. The corresponding sea-ice thickness losses for the mooring locations are 10 and 34 cm for MB5 and 20 and 2 cm for MB6, respectively. When compared to 2021 to 2023 and even 2013 to 2015, these values are within the lower range of δ*F*_h_ and sea-ice loss estimates for the eastern Eurasian Basin. This is to be expected because as the AW advances into the Arctic Ocean, it cools, resulting in less heat available for ventilation [e.g., (*11*)]. However, the presence of deep ventilation in the Amerasian Basin as far as 173.7°E (the MB6 mooring’s longitude) offers a remarkable indication of the eastward progression of atlantification.

The 2021-to-2023 MB7 mooring record, in contrast to the mooring MB5 and MB6 records in the central Makarov Basin, does not show any deep winter ventilation according to the winter survival of the near-surface temperature maximum, which is created by the summertime trapping of solar energy below the surface mixed layer [30- to 40-m depth; (*31*)] and lack of a cooling trend below the surface mixed layer (>60 m) in winter (Fig. 1E). Furthermore, the 5-year (2017 to 2023) record of the KAMS1 mooring shows a consistent pattern without ventilation of the halocline waters below the surface mixed layer, providing a longer context for the MB7 mooring record (Fig. 1E). However, both MB7 and KAMS1 mooring records show a clear Atlantic halocline (isotherm, −1.0°C)/AW layer shoaling (10.1 ± 0.3/4.1 ± 0.6 and 13.8 ± 1.2/5.8 ± 0.3 m per year, respectively), indicating that the region is undergoing preconditioning toward a shallower AW (and more accessible AW heat) and less stratified halocline (as evidenced by negative squared buoyancy frequency trends of −1.4 ± 0.2 and −0.7 ± 0.0 10^3^ s^−2^ per year in a 65- to 140-m layer at both mooring locations). This preconditioning is similar to what was observed during the 2000s in the eastern Eurasian Basin.

**Fig. 4.**
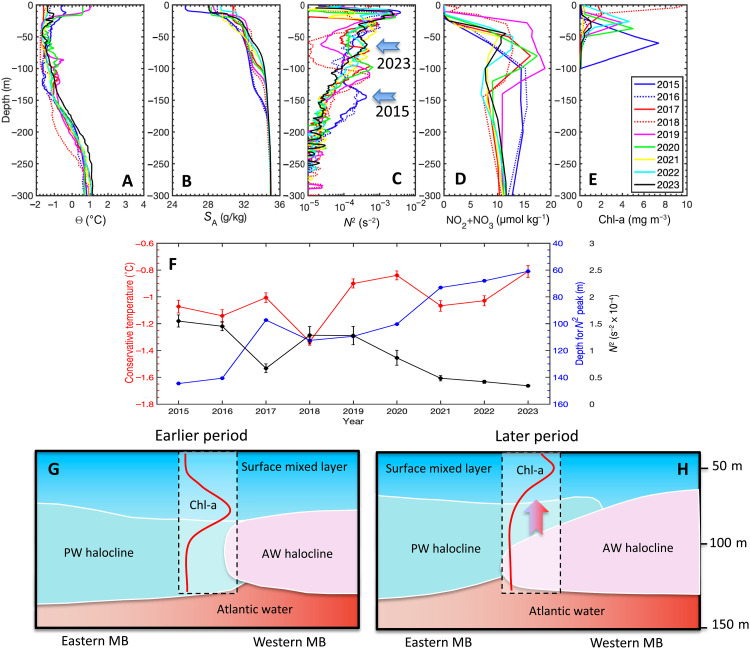
Physical and biogeochemical changes in the eastern Makarov Basin (MB) and East Siberian Sea in response to atlantification. (**A** to **E**) (Top) Vertical profiles of physical, chemical, and biological variables observed at station P (the position is indicated as * in Fig. 1E) in the summers of 2015 to 2023. (A) Potential temperature (θ, °C), (B) salinity (*S*, psu), (C) squared buoyancy frequency (*N*^2^, s^−2^), (D) nitrates (NO_2_ + NO_3_, μmol kg^−1^), and (E) total chlorophyll a (Chl-a, mg m^−3^). The arrows in (C) point to the position of the maximum of stratification (*N*^2^) in 2015 and 2023, which denotes the boundary between the PW (Pacific water) and AW halocline domains that shoals over time. (**F**) (Middle) Time series of water temperature (red); buoyancy frequency, which decreases in time, showing weakening of water column stability (*N*^2^, black); and depth of the maximum *N*^2^ (blue). Error bars show one standard error. (**G** and **H**) (Bottom) Diagram illustrating the environmental changes brought about by atlantification at station P. The vertical arrow in (H) shows that the Chl-a maximum shoaled from 2015–2016 (earlier period) to 2022–2023 (later period) in response to the incursion of Atlantic halocline waters, which forced nutrient-rich Pacific halocline water closer to the surface.

Systematic summer observations provide additional evidence for the increasing atlantification of the eastern Makarov Basin and East Siberian Sea since 2015 (Fig. 4). Consistent with the findings in (*32*) based on 2015-to-2017 observations, repeat 2015-to-2023 snapshot profiles and time series of water temperature and salinity show gradual warming and shoaling of AW and weakening of the stability of the halocline (~50 to 200 m), as indicated by the decreased values of the squared buoyancy frequency *N*^2^ (Fig. 4, A to C and F). Furthermore, the AW halocline associated with atlantification shoaled the maximum of *N*^2^, a marker of the boundary between the Pacific and Atlantic halocline domains, by around 70 m between 2015 and 2023 (Fig. 4, C and F). This process directly affected the shoaling of nutrient-rich waters into the euphotic zone, which may result in increased utilization of nutrients. The availability of nutrients controls the occurrence of summer surface blooms, as indicated by the shift of the chlorophyll a (Chl-a) maximum closer to the surface since 2015 (Fig. 4E) and schematically illustrated in Fig. 4, G and H [see also (*32*)]. Thus, even in this early preconditioning stage of atlantification in this part of the Siberian Arctic, physical changes had an immediate impact on the state of the local ecosystem.

## DISCUSSION

Changes associated with atlantification are extensive and fundamental for the state of the high-latitude climate system. The primary impact has been the rapidly diminishing sea ice in the Siberian Arctic over the past decade (Fig. 1, A and B).

**Fig. 5.**
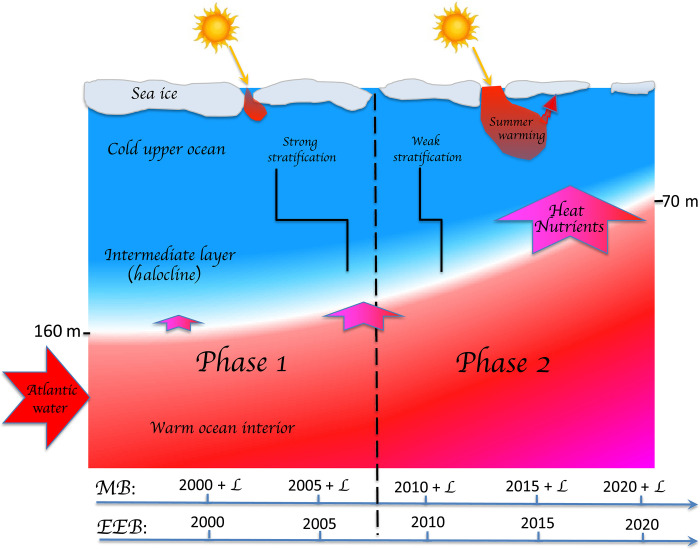
Two phases of atlantification of the Arctic Ocean. Phase 1: warming of the ocean interior and AW/nitricline shoaling. The halocline remains relatively strong, preventing deep ventilation. Phase 2: halocline weakened enough to allow deep ventilation. The shoaling of Atlantic water from ~150 m in the early 2000s to up to 70 m in recent years in the eastern Eurasian Basin has coincided with the seasonal disappearance of the halocline, which enhances upward heat and nutrient transport from the ocean interior to the upper ocean, causing fast sea-ice loss and an increase in primary production. A positive ocean-heat/ice-albedo feedback mechanism is triggered, amplifying the path of atlantification. The eastward progression of atlantification into the Makarov Basin (MB) toward Alaska lags the eastern Eurasian Basin (EEB) by L years.

Here, we used mooring and satellite data to demonstrate the advancement of atlantification into the Siberian Arctic Ocean and its ramifications for the state of the upper ocean, sea ice, and air-sea interactions (Fig. 5). In particular, we show that the winter ventilation of the halocline in the eastern Eurasian Basin resulted in more than twofold rate in sea-ice loss caused by oceanic heat fluxes as compared to the 2010s. The transition of the Makarov Basin to conditions similar to those observed in the eastern Eurasian Basin 5 to 7 years ago is another critically important finding (this lag is depicted by L in Fig. 5). The powerful ocean-heat/ice-albedo feedback mechanism is the primary cause of these changes (phase 2 of atlantification in Fig. 5). Deep ventilation and weak stratification increase upward AW heat fluxes, which promotes the wintertime suppression of sea-ice formation and subsequent more effective summertime reduction of sea ice by an ice-albedo feedback. This complex process was the key to establishing the diminished sea-ice cover in the Siberian Arctic in recent years. In contrast, no deep ventilation of AW heat was found in the eastern parts of the Makarov Basin and East Siberian Sea. Shoaling of the AW and halocline, however, indicates that the eastern Siberian Arctic Ocean is experiencing a preconditioning phase (phase 1 of atlantification in Fig. 5) similar to that found in the western Siberian Arctic Ocean in the 2000s. This ongoing transition not only mirrors earlier changes but also sets the stage for broader ecosystem impacts. While it was identified that the intrusion of Atlantic-origin water into the Chukchi Plateau is associated with biogeochemical impacts (*33*), our analysis reveals that these physical changes—particularly AW shoaling, halocline weakening, and seasonal variability in the Atlantic/Pacific halocline front—are establishing conditions for halocline stability disruptions and increased AW penetration. These atlantification-related physical changes have important ecological implications.

The lingering question pertains to the future trajectory of atlantification: Will it persist in its eastward progression or not? Climate models serve as the sole available means to inform us, yet they unfortunately diverge in their projections of future atlantification (*21*), partly due to their challenges in accurately reproducing Arctic Ocean hydrography (*34*) and surface climate (*35*). They often exhibit significant biases in AW properties, inadequately depict the AW circulation, and substantially under- or overestimate the amount of heat transported into the Arctic. These limitations are partly attributed to low resolution (*36*), poorly constrained mixing schemes (*37*), and a strong decoupling between the upper and deep ocean, such as the lack of deep ventilation (*34*). Modeling results in (*21*) indicate that atlantification will not advance into the Amerasian Basin due mainly to enhanced surface freshening. However, an unrealistic vertical and horizontal distribution of this freshwater (*38*), a general fresh bias resulting in overestimated freshening (*39*), and a too-deep and too-cold AW layer (*34*) are all factors that could potentially result in an underestimation of the rate of atlantification in global climate models from the CMIP-6 experiment, as also hypothesized in (*21*). Model stratification biases can also alter the pathway of AW where the Lomonosov Ridge meets the continental slope by influencing the vorticity constraints on topographic flow, thereby affecting the position of the atlantification front. Improving these models is therefore necessary before we can have more certainty in the future eastward progression of atlantification.

Despite their limitations, climate models do converge on certain overarching Arctic changes that are recognized to affect atlantification. For instance, they consistently forecast AW warming (*40*) and heightened heat transport (*37*). In addition, they consistently simulate a reduction of sea-ice area and thickness, which, along with strengthened winds, likely results in an increase in atmosphere-ocean momentum transfer (*41*). This is also expected to drive stronger surface ocean currents, increased ice drift, and intensified vertical mixing. Given our mechanistic understanding, it is probable that these trends will contribute to the advancement of atlantification, although the hydrographic biases limit our ability to predict this accurately.

Meanwhile, the Siberian Arctic Ocean, east of the Lomonosov Ridge, is already exhibiting signs of atlantification, characterized by enhanced upper ocean ventilation rates and significant regional sea-ice losses. These changes are expected to profoundly affect the local ecological systems, influencing primary production and species distribution in the Pacific Arctic, with uncertain implications for the broader food web [e.g., (*42*)].

## MATERIALS AND METHODS

In this study, we use Arctic atmospheric, sea-ice, and oceanic data.

### Satellite sea-ice concentration

The satellite archive used in this study includes Advanced Very High-Resolution Radiometer global daily sea surface temperature and sea-ice concentration from 1981 to 2022 with 0.25° by 0.25° spatial resolution (https://psl.noaa.gov/data/gridded/tables/sst.html). Figure 1 (A and B) uses daily sea-ice concentration maps from AMSR-E (Advanced Microwave Scanning Radiometer for EOS) and AMSR2 (*43*). The daily maps are available from the University of Bremen, Institute of Environmental Physics (https://seaice.uni-bremen.de).

### Atmospheric data

Monthly surface air temperatures during 1979 to 2021 used in this study are from the European Centre for Medium-Range Weather Forecasts reanalysis ERA5, downloaded from www.ecmwf.int/en/forecasts/dataset/ecmwf-reanalysis-v5 (*44*). The horizontal resolution of the data is 0.25°.

### Oceanic data

#### Mooring observations

Our analysis uses the collection of instrumental observations of ocean temperature from five moorings distributed in the Siberian Arctic Ocean (Fig. 1F). Moorings MB1, MB5, MB6, and MB7 maintained by the Nansen and Amundsen Basins Observational System program were deployed from September 2021 through September 2023 and provided conductivity-temperature-depth (CTD) profiles from McLane Moored Profilers (MMP) for the depth range of ~40 to 1000 m complemented by fixed-depth observations by SBE-37 CTD for the depth range of 25 to 40 m. Mooring KAMS1 maintained by the Korean Polar Research Institute (KOPRI) was deployed from August 2017 through September 2023 and provided CTD time series made by SBE-37 and temperature time series provided by several SBE-56 from fixed depths.

The MMP temperature- and conductivity-calibrated measurement accuracies are ±0.002°C and ±0.002 mS/cm, respectively. The MMP sampled a vertical profile along a mooring line once per 2 days at a speed of ~25 cm/s with a sampling period of 0.5 s; therefore, the data had a vertical spacing of ~12 cm. SBE-37 and SBE-56 provided 15-min interval records with measurement accuracies of ±0.002°C and ±0.003 mS/cm for SBE-37 temperature and conductivity, respectively, and ±0.002°C for SBE-56 temperature. At MB1, mooring site observations began in August 2002, thus providing invaluable long-term measurements (Fig. 1, D and E). Nansen and Amundsen Basins Observational System mooring data used in this study are available in (*45*, *46*). Mooring KAMS1 data are available from the Korea Polar Data Center (KPDC) website (https://dx.doi.org/doi:10.22663/KOPRI-KPDC-00002462.1).

#### Ocean temperatures, salinities, and biogeochemical parameters from ship-borne observations

Our analysis uses instrumental snapshot observations of ocean temperature, salinity, and chemical and biological parameters from the P station in the eastern East Siberian Sea made annually from 2015 through 2023 (coordinates: 75.1701^o^N, 179.9658^o^W; see the location marked by * in Fig. 1F). These data were obtained from the summer (August) expeditions of the Korean icebreaker IBR/V *Araon* (ARA06B to ARA14B). Vertical profiles of ocean temperature and salinity were measured with an SBE911plus CTD profiler and processed to produce 1-m averaged downcast profiles. The accuracies for temperature and conductivity are ±0.001°C and ±0.003 S/m, respectively. Salinity data were calibrated with the bottle-sampled seawater salinity measured by an autosalinometer (Guildline Co., model 8400B) (*47*). Seawater samples for nutrient and Chl-a measurements were collected using Niskin bottles at surface and discrete depths chosen on the basis of CTD profiles. Nutrients, including nitrite + nitrate (NO_2_ + NO_3_), phosphate (PO_4_), ammonium (NH_4_), and silicic acid [Si(OH)_4_], were measured on board using a four-channel continuous autoanalyzer (QuAAtro, Seal Analytical, Germany). Nutrient reference materials for seawater (lot. no. BV, KANSO Technos Co., Ltd., Osaka, Japan) were measured along with standards for every batch of runs to assess the accuracy and reproducibility. The precision values for NO_2_ + NO_3_, PO_4_, and Si(OH)_4_ were ±0.14, ±0.02, and ±0.28 μmol kg^−1^, respectively. Seawater samples for Chl-a were filtered through 47-mm GF/F filters and were then extracted with 90% acetone for 24 hours. The concentration of Chl-a was measured using a fluorometer (Trilogy, Turner Designs, US) (*48*). The CTD, nutrient, and Chl-a data at the P station are available from the KPDC website (https://dx.doi.org/doi:10.22663/KOPRI-KPDC-00002462.1).

### Definition of heat content *Q*

We used the heat content *Q* (J/m^3^) to quantify winter heat ventilation and associated divergent heat fluxes and equivalent sea-ice losses in the upper ocean. It is defined as

$$Q=\rho_{w}c_{P}\left(\theta -\theta_{\text{fr}}\right)$$ (1)

where θ is the potential temperature, θ_fr_ is the freezing temperature, ρ_w_ is the water density, and *c*_P_ is the specific heat of seawater. In physical terms, *Q* may be understood as the relative heat content, measuring how much heat needs to be removed to form ice crystals from the water column at particular salinity and pressure.

### Definition of divergent heat flux δ*F*_h_

Following (*49*), let us consider a simple model that describes changes in the heat budget within a unit-area water column of the upper ocean within a depth range of thickness *H* = *z*_2_ − *z*_1_, where *z*_1_ and *z*_2_ are upper and lower depth limits. The vertically integrated heat content $\hat{Q}$ for this box is a combination of lateral and vertical advection and divergence of turbulent heat fluxes

$$\bar{\delta \theta }\approx \delta t\left[-U\frac{\partial \bar{\theta }}{\partial x}-W\frac{\partial \bar{\theta }}{\partial z}+\frac{\partial }{\partial x}K_{H}\frac{\partial \bar{\theta }}{\partial z}+\frac{1}{\rho c_{P}H}\delta F_{h}\right]$$ (2)

In Eq. 2, the overbar denotes the vertical mean within *H*, $\delta \bar{\theta }$ is the change in potential temperature θ over a period of time δ*t*, ρ is the water density, *U* is the along-trajectory horizontal velocity, and *W* is the vertical velocity. The last two terms in Eq. 2 describe the divergence of lateral and vertical heat fluxes. The coefficient *K*_H_ is the lateral diffusivity, and δ*F*_h_ is the difference of diapycnal heat fluxes *F*_h_ at *z*_2_ and *z*_1_. Typically, *F*_h_ is calculated as Fh=−ρcPKz∂θ∂z with *K_z_* being the diapycnal diffusivity; however, here, we estimate δ*F*_h_ from changes in vertically integrated heat content $\hat{Q}$, not via measured ∂θ/∂*z* and estimated *K_z_*.

The lateral advection and diffusion of heat (the first three terms in Eq. 2 on the right-hand side) do not significantly contribute to the observed winter ventilation of the halocline (*12*, *49*). Here, we further support arguments presented in these publications. For example, we found that the cross-slope heat exchanges at various mooring sites varied out of phase below the depth of the surface winter ventilation (not shown). Moreover, seasonal upwelling or downwelling of the AW heat cannot explain the observed changes. First, we found seasonal fluctuations discussed here at deep (>2500 m) water moorings, which are far away from the area that may be affected by upwelling. Second, Ekman pumping (wind-driven vertical advection) is too weak to explain the observed vertical displacements and the changes in θ (*49*). Considering the role of the along-slope advection, a water parcel’s time to travel eastward should result in out-of-phase seasonal variations. For example, we estimate that the water mass travel time from mooring site MB1 to MB5 (at an average speed of ~3 cm/s) and farther eastward from mooring site MB5 to MB6 (at an average speed of ~1.5 cm/s) will take ~270 and 170 days, respectively. If advection is the primary mechanism generating seasonal signals at these mooring locations, we should expect out-of-phase seasonal fluctuations. This is not the case. For every mooring and freely drifting buoy record, over every year and every location [(*12*, *49*) and this study], we observe in-phase upper ocean seasonal variations in θ. These are strong arguments in favor of the validity of our approach.

Note that the estimates of δ*F*_h_ used in our study are flux differences; therefore, our inferred estimates of heat fluxes represent a lower bound on the total heat flux. Because of additional nondivergent heat exchanges, the total heat fluxes may exceed these estimates. The divergence of vertical heat flux δ*F*_h_ is estimated using seasonal trends of vertically integrated heat content $\hat{Q}$ during the winter cooling period. The depth of the winter ventilation *H*_vent_ is the depth below which changes in $\hat{Q}$ are not related to surface processes. Hence, increasing the depth of integration beyond *H*_vent_ for the purposes of calculating $\hat{Q}$ would not result in a statistically significant change in $\hat{Q}$. The sensitivity of such estimates to the selection of *H*_vent_ is explored in (*12*).

### Characterizing stratification

Stratification in the upper ocean is quantified using the buoyancy (Brunt-Väisälä) frequency, which is calculated as follows

$$N={\left[-g/\rho_{o}\frac{\partial \rho_{w}}{\partial z}\right]}^{1/2}$$ (3)

where *g* is the gravitational acceleration, and ρ_o_ is the mean water density. An increase in *N* indicates increasing stratification (typically precluding mixing), whereas a decrease in *N* signifies a less stratified ocean better preconditioned for mixing.

### Estimating statistical significance of trends

The least-squares best-fit method was used to assess trends in the SAT. Student’s *t* statistic with a 95% confidence interval is used to assess the statistical significance of trends (*50*). The high horizontal resolution of the ERA5 reanalysis data prevented presenting all stippling signs (crosses) used for identifying nonsignificant trends in Fig. 3 and figs. S2 and S3; stippling was therefore shown every third point in the latitudinal direction and every 10th (60th) point at 75°N (89°N) in the longitudinal direction.
