# Supplementary material for: Atlantification advances into the Amerasian Basin of the Arctic Ocean
Source: Sci Adv. 2025 Feb 21;11(8):eadq7580. doi: 10.1126/sciadv.adq7580 (PMC11844727; doi:10.1126/sciadv.adq7580)
Supplement: Supplementary file 1 — Figs. S1 to S3 [file sciadv.adq7580_sm.pdf]

Science Advances
AAAS

Supplementary Materials for

Atlantification advances into the Amerasian Basin of the Arctic Ocean

Igor V. Polyakov *et al.*

Corresponding author: Igor V. Polyakov, ivpolyakov@alaska.edu

Sci. Adv. **11**, eadq7580 (2025)
DOI: 10.1126/sciadv.adq7580

This PDF file includes:

Figs. S1 to S3

Supplementary Figures

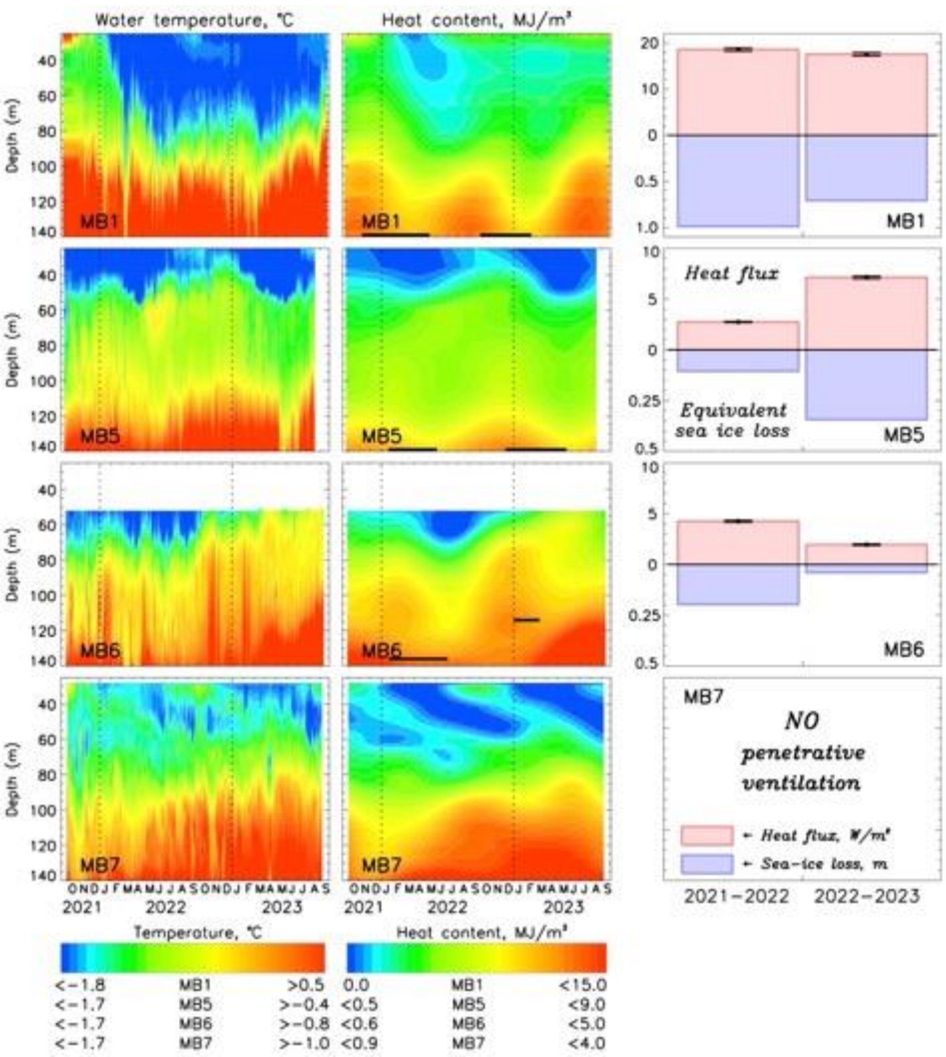

Figure S1: **Sea ice losses due to oceanic heat transfers along the Siberian slope in 2021–2023**.

(Left and middle) 25-140m layer depth versus time of water temperature and annual component of heat content *Q*. Annual components are obtained via band-pass filtering using wavelet transforms. Maxima and minima of vertically integrated *Q* were used to define the boundaries of winter ventilation seasons. Horizontal black segments identify the depth of seasonal ventilation; start and end dates of the ventilation seasons are shown by the ends of these segments.

(Right) Slope of trends in *Q* within these boundaries defines the rate of change of *Q* in time, which is equivalent to the divergent heat flux F_h (red bars; errors are shown by black segments). Equivalent sea ice thickness losses are shown as blue bars.

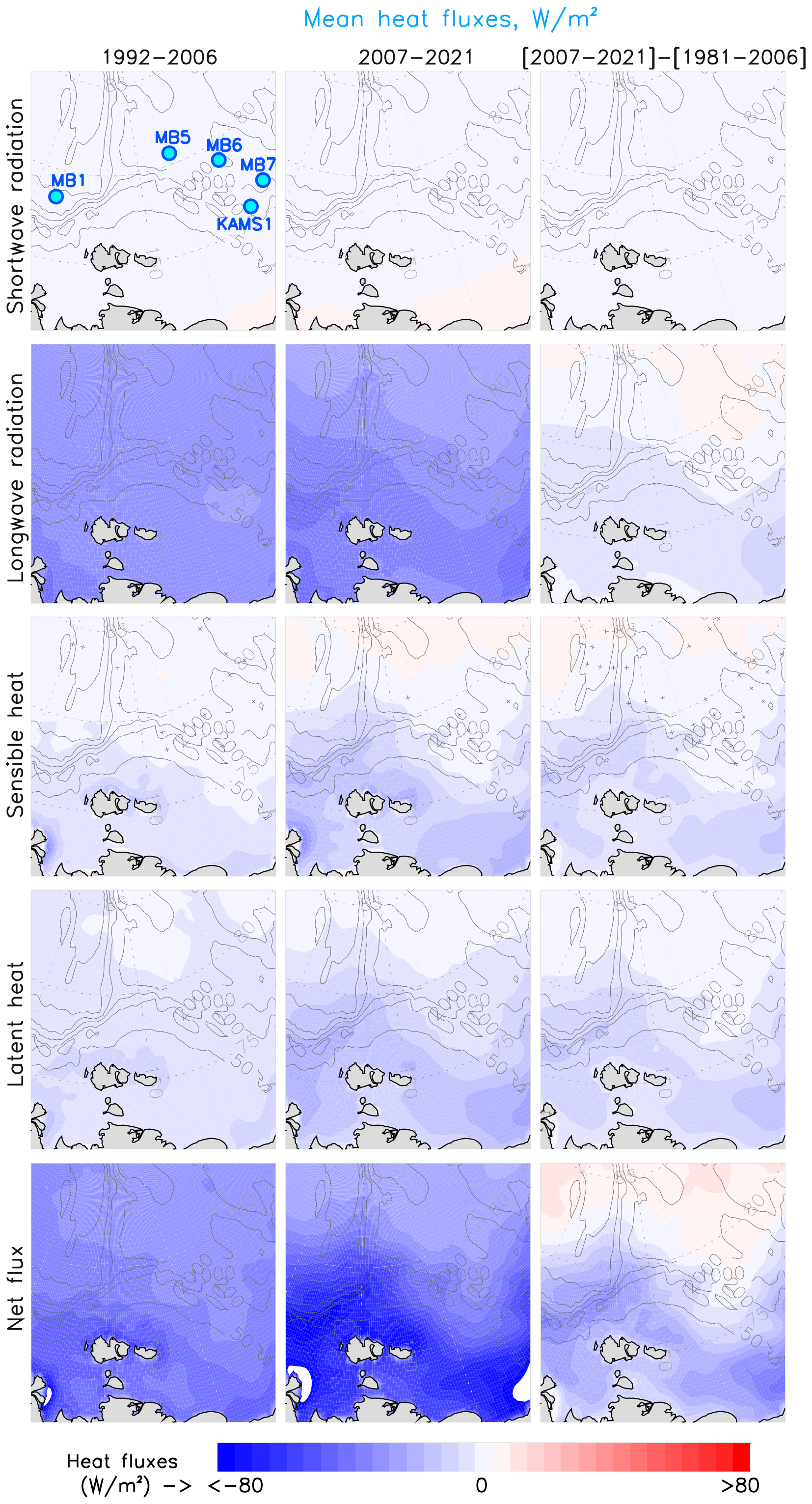

Figure S2: Mean October 15 – November 15 air-sea heat fluxes over the Siberian Arctic Ocean. Negative fluxes indicate the heat flux from the ocean to the atmosphere averaged over 1992-2006 (left), 2007-2021 (middle), and their difference (right). Stippling shows areas with statistically insignificant means. Bottom topography is shown by grey lines (m). Mooring locations are shown in the top left panel.

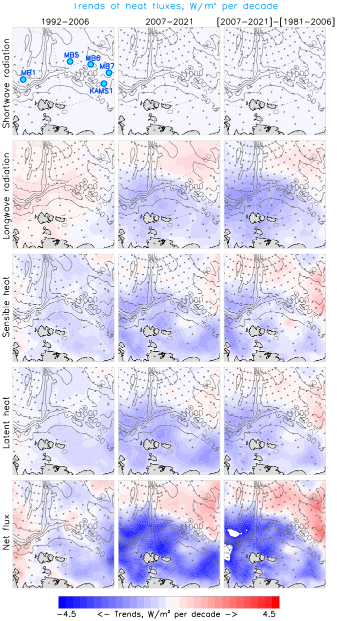

Figure S3: Trends in October 15 – November 15 air-sea heat fluxes over the Siberian Arctic Ocean. Trends are for 1992-2006 (left), 2007-2021 (middle), and their difference (right). Negative trends indicate an increase in the heat flux from the ocean to the atmosphere. Stippling shows areas with statistically insignificant trends (typically, where trends are weak). Bottom topography is shown by grey lines (m). Mooring locations are shown in the top left panel.
